# Th1 Stimulatory Proteins of *Leishmania donovani*: Comparative Cellular and Protective Responses of rTriose Phosphate Isomerase, rProtein Disulfide Isomerase and rElongation Factor-2 in Combination with rHSP70 against Visceral Leishmaniasis

**DOI:** 10.1371/journal.pone.0108556

**Published:** 2014-09-30

**Authors:** Anil Kumar Jaiswal, Prashant Khare, Sumit Joshi, Pramod Kumar Kushawaha, Shyam Sundar, Anuradha Dube

**Affiliations:** 1 Division of Parasitology, CSIR-Central Drug Research Institute, Lucknow, India; 2 Department of Medicine, Institute of Medical Sciences, Banaras Hindu University, Varanasi, India; Federal University of São Paulo, Brazil

## Abstract

In visceral leishmaniasis, the recovery from the disease is always associated with the generation of Th1-type of cellular responses. Based on this, we have previously identified several Th1-stimulatory proteins of *Leishmania donovani* -triose phosphate isomerase (TPI), protein disulfide isomerase (PDI) and elongation factor-2 (EL-2) etc. including heat shock protein 70 (HSP70) which induced Th1-type of cellular responses in both cured *Leishmania* patients/hamsters. Since, HSPs, being the logical targets for vaccines aimed at augmenting cellular immunity and can be early targets in the immune response against intracellular pathogens; they could be exploited as vaccine/adjuvant to induce long-term immunity more effectively. Therefore, in this study, we checked whether HSP70 can further enhance the immunogenicity and protective responses of the above said Th1-stimulatory proteins. Since, in most of the studies, immunogenicity of HSP70 of *L. donovani* was assessed in native condition, herein we generated recombinant HSP70 and tested its potential to stimulate immune responses in lymphocytes of cured *Leishmania* infected hamsters as well as in the peripheral blood mononuclear cells (PBMCs) of cured patients of VL either individually or in combination with above mentioned recombinant proteins. rLdHSP70 alone elicited strong cellular responses along with remarkable up-regulation of IFN-γ and IL-12 cytokines and extremely lower level of IL-4 and IL-10. Among the various combinations, rLdHSP70 + rLdPDI emerged as superior one augmenting improved cellular responses followed by rLdHSP70 + rLdEL-2. These combinations were further evaluated for its protective potential wherein rLdHSP70 + rLdPDI again conferred utmost protection (∼80%) followed by rLdHSP70 + rLdEL-2 (∼75%) and generated a strong cellular immune response with significant increase in the levels of iNOS transcript as well as IFN-γ and IL-12 cytokines which was further supported by the high level of IgG2 antibody in vaccinated animals. These observations indicated that vaccine(s) based on combination of HSP70 with Th1-stimulatory protein(s) may be a viable proposition against intracellular pathogens.

## Introduction

Visceral leishmaniasis (VL) or Kala-azar, one of the most neglected tropical diseases, is caused by three leishmanial species, *L. donovani*, *L. infantum* and *L. chagasi* depending on the geographical area. *L. infantum* infects mostly children and immunosuppressed individuals whereas *L. donovani* infects individuals of all age groups. VL is endemic in 62 countries, with a total of 200 million people at risk and estimated 500,000 new cases of VL each year worldwide [1], [2]. In India, kala-azar has been reported mostly from the states of Bihar, Assam, West Bengal and Eastern Uttar Pradesh. Currently available treatment for VL is highly unsatisfactory due to their toxicities and side effects. Besides, there are several reports of unresponsiveness to pentavalent antimonials (SbV) in recent years [3]–[5]. In a survey in Bihar, there were a record alarming 100,000 cases of VL, of which 10,000 are unresponsive to SbV [6]. Therefore, this situation demands for an alternative control strategy posing an urgent need of a safe and effective vaccine, although, the development of an effective *Leishmania* therapeutic/prophylactic vaccine has been a challenge. Parasitic antigens that induce a significant level of immune response have been primarily associated with the identification of proteins that may be used for vaccine development. Numerous studies showed antigens from different *Leishmania spp*. were able to stimulate IFN-γ and IL-12 expression levels [7]–[11], the signatures for Th1 type of immune response [7]–[9]. However, among these, only IFN-γ represents the key cytokine involved in the activation of macrophages for the killing of *Leishmania* parasites [10], [11]. Therefore, the Th1 feature of the immune response could be exploited as vaccine candidates. Till date besides killed or live-attenuated parasites, several *Leishmania* antigens from different species either as DNA or as protein vaccines were tested against VL with different level of success. These observations provide sufficient evidences that a vaccine against VL is feasible.

Based on this rationale, several potential immunogenic antigens from *L. donovani* were identified through proteomics inducing Th1 type immune response in the PBMCs of cured/endemic *Leishmania* patients [12]–[14]. Heat shock protein 70 (HSP70) was one amongst them identified as potential T-cell stimulatory protein along with Aldolase, Enolase, P45, Protein Disulfide Isomerase (PDI), Triose Phosphate Isomerase (TPI) and Elongation Factor-2 (EL-2). The Heat Shock Proteins (HSPs) are highly conserved molecules and present in all eukaryotes and prokaryotes particularly localised in sub cellular region of parasites [15]. HSPs play many important roles like folding, assembly, intracellular localization, secretion, and degradation of many proteins, hence HSPs has been also termed as molecular chaperones [16]. Many studies favor the involvement of chaperones in many immunological processes such as in assembly of immunoglobulins, T-cell receptors, and major histocompatibility complex (MHC) molecules and participate in antigen processing and presentation pathways [17]–[20]. The usage of HSP70 as a potent adjuvant in immunotherapy of cancers and other infectious diseases has been well documented [17], [18], [21]–[24]. The adjuvant effect of HSP70 has been demonstrated after immunization with *Plasmodium* peptides [21], *L. infantum* [22] and *L. donovani* [23], [24].

In most of the studies, immunogenicity of HSP70 of *L. donovani* was assessed in native condition [23], [25], [26]. Herein, we developed recombinant HSP70 of *L. donovani* and further tested for its potential to stimulate immune responses in lymphocytes of cured *Leishmania* infected hamsters as well as in the peripheral blood mononuclear cells (PBMCs) of cured patients of VL either individually or in combination with recombinant proteins of *L. donovani* viz. PDI, TPI and EL-2 which were identified earlier as potent Th1 stimulatory proteins through immuno-proteomics and were found to be protective to hamsters against *Leishmania* challenge [13],[14],[27]–[29]. Further, we examined the ability of above mentioned combinations, to protect the hamsters against *L. donovani* challenges when administered as recombinant protein vaccine.

## Materials and Methods

### Ethics Statement

Experiments on the animals (hamsters) were performed following the approval of the protocol and the guidelines of Institutional Animal Ethics Committee (IAEC) of the CDRI which is adhered to National Guideline of CPCSEA (Committee for the Purpose of Control and Supervision on Experiments on Animals) under the Ministry of Environment and Forest, Government of India. The approval reference number 154/10/Para/IAEC dated 04.10.10. The protocol and study with patients was approved by the Ethics committee of the Kala-azar Medical Research Centre, Muzaffarpur (Protocol # EC- KAMRC/Vaccine/VL/2007-01) and written informed consent was obtained from patients before enrolment to this study. All the human subjects underwent clinical examination by a local physician for leishmanial and other possible infections.

### Host and Parasites

Golden hamsters (*Mesocricetus auratus*, 45–50 g) of either sex were used for all experimental work. They were maintained in climatically controlled room of animal house facility of Central Drug Research Institute (CDRI) and were fed with standard rodent food pellet (Lipton India Ltd.) and water *ad libitum*. The *L. donovani* strain (2001) was procured from Institute of Medical Sciences, BHU, Varanasi and was maintained *in vitro* as described elsewhere [30]. *L. donovani* promastigotes were grown in RPMI-1640 (Sigma-Aldrich, USA) at 26°C in 75 cm^2^ culture flasks (Nunc, Denmark) [31]. Parasites were also maintained in hamsters through serial passage i.e. from amastigote to amastigote in order to maintain the virulence of parasite [31].

### Preparation of soluble *L. donovani* promastigote antigen

Soluble *L. donovani* (SLD) promastigote antigen was prepared as per the method described elsewhere [12], [31]. Briefly, log phase promastigotes (10^9^) were harvested from 3 to 4 days old culture and washed 3 to 4 times with cold phosphate-buffered saline (PBS) and resuspended in PBS containing protease inhibitor cocktail (PIC) (Sigma-Aldrich, USA) and subjected to ultrasonication and centrifugation at 40,000×g for 30 min. The protein content of the supernatant was estimated [32] and stored at −80°C.

### Cloning, expression, purification and immunoblotting of recombinant protein

Genomic DNA was isolated from *L. donovani* promastigotes according to protocol described elsewhere [27]. HSP70 gene was amplified using specific forward primer- 5′-GGATCCATGACATTCGACGGCGCCATG-3′and reverse primer-5′ GAATTCGTCCT TGCTCAGCCGGCCCT-3′ which were designed using *L. major* HSP70 gene sequence (accession no. M36675.1) as template. The restriction sites for *BamHI* and *EcoRI* are under lined in forward and reverse primers respectively. The HSP70 gene was amplified using Taq DNA Polymerase (Bangalore Genei, India) lacking 3′–5′ exonuclease activity in a Thermocycler (Bio-Rad, USA). Amplified PCR product was first cloned into the pTZ57R/T vector and then into bacterial expression vector - pET28a^+^. Further, cloned insert was sequenced and sequence submitted to the National Center for Biotechnology Information (Accession no. HQ011382). The clone containing the construct (rLdHSP70 + pET28a^+^) was used to express the *L. donovani* HSP70 in *E. coli Rosetta* strain following induction with 1.0 mM isopropyl-β-D-thiogalacto-pyranoside (IPTG) at 37°C. The recombinant protein was purified using Ni-NTA agarose column (Qiagen Valencia, CA), following standard procedures and lysis buffer as 50 mM Tris, 300 mM NaCl, 10 mM imidazole and 10% glycerol (pH 8.0). Further, the purified protein was analyzed by 12% SDS–PAGE, stained with coomassie brilliant blue stain (Sigma-Aldrich, USA) and the protein content was estimated by the Bradford method using bovine serum albumin (BSA) as standard. The lipopolysaccharides (LPS) content of the recombinant proteins was measured by Limulus amoebocyte lysate test (QCL-1000, Lonza).

The purified rLdHSP70 protein was used for raising antibodies in Swiss albino mice. Mice were immunized with 50 µg of rLdHSP70 in combination of Freund's complete adjuvant following two booster doses of 25 µg of rLdHSP70 after 15 days (two weeks interval) in combination of Freund's incomplete adjuvant and blood was collected for serum 8 days after the last immunization. For immunoblotting experiment, purified rLdHSP70 protein and SLD were resolved on 12% SDS–PAGE and transferred to nitrocellulose membrane using a semi-dry blot apparatus (Amersham) [33]. Similarly, rLdHSP70 was transferred to another set of membrane parallely. After overnight blocking in 5% BSA, both the membranes were incubated with antiserum to the rLdHSP70 proteins (dilution of 1∶4000) and serum (1∶100) from VL patient's respectively for 2 h at room temperature (RT) followed by three times washing with PBS containing 0.5% Tween-20 (PBST). Further, the blot treated with antiserum to rLdHSP70 was incubated with secondary antibody-goat anti-mice IgG HRP conjugate (1∶10,000) (Bangalore Genei, India) and blot treated with patient's serum was incubated with secondary antibody- mouse anti-human IgG biotin conjugate (1∶1000) followed by HRP conjugated streptavidin (1∶500) (BD Pharmingen, USA) for 1 h at RT. Both the blots were developed by using diaminobenzidine + imidazole + H_2_O_2_ (Sigma-Aldrich, USA).

However, cloning, expression and purification of *L. donovani* Triose phosphate Isomerase (LdTPI) Protein Disulfide Isomerase (LdPDI) and Elongation Factor 2 (LdEL-2) were done earlier [27]–[29]. Further, all these recombinant proteins were purified following the reported protocol.

### Treatment of *L. donovani* infected hamsters and isolation of mononuclear cells from lymph node

Approximately 30 hamsters were infected with 10^7^ amastigotes intracardially and checked for parasite burden as described elsewhere [13]. Animals harbouring >20–25 amastigotes/100 macrophage cell nuclei were then treated with miltefosine (SynphaBase, Switzerland) given orally @ 40 mg/kg bodyweight for 5 days. Animals were reassessed for complete cure by splenic biopsy performed on day 30 post treatment. Mononuclear cells were separated from lymph nodes of cured, infected as well as from normal hamsters as per the protocol of Garg et al. [30]. Finally a suspension of 10^6^ cells/ml was made in cRPMI (Sigma-Aldrich, U.S.A) which were further utilized for lymphoproliferative assay and for the estimation of NO production.

### Isolation of peripheral blood mononuclear cells (PBMCs) from different groups of human patients

The study groups for human samples were as follows:

1. Eight cured patients (5 males +3 females, age range 5–32 years) from hyper-endemic areas of Bihar. All the patients had received complete course of miltefosine treatment and had recovered from VL. Diagnosis was established in all cases by demonstration of parasites in splenic aspirates and found negative at the time of study.
2. Eight endemic household contacts (6 males +2 females, age ranging from 8 to 35 years) who neither showed clinical symptoms nor received any treatment for Kala-azar.
3. Eight infected patients (4 males +4 females, age range 5–49 years) showing clinical symptoms of Kala-azar.
4. Eight normal healthy donors (4 males +4 female, age range 25–32 years) from non-endemic areas, without any history of leishmaniasis, served as negative control.

Heparinised venous blood (10 ml each) was collected from all study subjects and peripheral blood mononuclear cells (PBMCs) were isolated from blood by Ficoll Hypaque density gradient centrifugation (Histopaque 1077, Sigma-Aldrich, USA) as described by Garg et al [30]. A final suspension of 1×10^6^ cells/ml was made in cRPMI (Sigma-Aldrich, USA) after determining cell viability by trypan blue (Sigma-Aldrich, USA) staining method.

### Vaccination with rLdHSP70 alongwith its various combinations (rLdHSP70 + rLdPDI, rLdHSP70 + rLdTPI, rLdHSP70 + rLdEL-2) in hamsters

Eleven groups of hamsters containing 15–20 animals per group, were taken for the study, wherein groups 1–4 served as controls i.e.- group-1, non-vaccinated and unchallenged (normal control); group-2, non-vaccinated and challenged (infected control); group-3, BCG alone and group-4 rLdHSP70 alone. The animals of group 5-8 were vaccinated intradermally (i.d.) with a dose of 50 µg/50 µl of either recombinant proteins rLdHSP70/rLdPDI/rLdTPI/rLdEL-2 along with equal volume of 0.1 mg per animal BCG respectively and group 9-11 were vaccinated with combinations viz. rLdHSP70 + rLdPDI, rLdHSP70 + rLdTPI, rLdHSP70 + rLdEL-2. After fourteen days a booster dose of half of the amount of recombinant proteins along with BCG was given i.d. route to all the hamsters of Group 5–8, only BCG to the animals of group-3, only rLdHSP70 to group-4. Similarly all the hamsters of group 9–11 were vaccinated with booster dose of their respective combinations. After 21 days the hamsters of Groups 2–11 were challenged intracardially (i.c.) with metacyclic promastigotes (10^7^) of *L. donovani*. After post challenge (p.c), on the day 45, 60, 90 and 120, three to four hamsters from each group were sacrificed for the assessment of parasitological and immunological (cytokines by real-time PCR and antibody level by ELISA and lymphoproliferation and NO production by earlier mention methods) progression of VL. The impression smears of different organs, such as spleen, liver, and bone marrow of experimental animals were made, and the criteria for the assessment of parasitic burden was based on the counting of the number of amastigotes per 100 cell nuclei in each organ. The percentage of inhibition (% inhibition) of parasite multiplication was calculated in comparison with the non-vaccinated control using the following formula: % inhibition  =  (number of amastigote count from infected control - number of amastigote from the vaccinated group/number of parasite count from infected control) ×100. Animals of all the groups were given proper care and observed for their survival period.

### Immunological assays

#### Delayed type hypersensitivity (DTH)

DTH was performed by injecting 50 µg/50 µl of SLD in PBS via i.d route into one footpad and PBS alone into another footpad of each of the vaccinated and unvaccinated controls. The response was evaluated 48 h later by measuring the difference in footpad swelling between the two, with and without SLD for each animal [8].

#### Lymphocyte proliferation and nitric oxide assay

These assays were carried out as per the protocol described by Garg et al [34] with slight modifications. PBMCs and mononuclear cells (1×10^6^ cells/ml) of cured/endemic/infected patients as well as cured/normal/infected hamsters respectively, were cultured in 96-well flat bottom tissue culture plates (Nunc, Denmark). About 100 µl of predetermined concentration of mitogens phytohaemagglutinin (PHA, 10 µg/ml Sigma-Aldrich, USA) for patient's PBMCs, concanavalin A (ConA, 10 µg/ml, Sigma-Aldrich, USA) for hamster's mononuclear cells, as well as rLdHSP70, rLdPDI/rLdTPI/rLdEL-2 and combinations (rLdHSP70 + rLdPDI, rLdHSP70 + rLdTPI, rLdHSP70 + rLdEL-2) and SLD (10 µg/ml each) were added to the wells in triplicate. Wells without stimulants served as blank controls. PHA and ConA were served as positive control for proliferation in human patients PBMCs and hamster's lymphocytes, respectively. Cultures were incubated at 37°C in a CO_2_ incubator with 5% CO_2_ for 3 days in case of mitogens as control, and for 5 days in case of test antigens (i.e. recombinant proteins). Eighteen hours prior to termination of experiment, 50 µl of XTT (Roche Diagnostics) was added to 100 µl of supernatants of each well and absorbance was measured at 480 nm with 650 nm as reference wavelength.

Similarly nitric oxide (NO) estimation was done using standard control mitogens (LPS, 10 µg/ml). NO level was assessed using Griess reagent (Sigma-Aldrich, USA) in the culture supernatants of naive hamster peritoneal macrophages [30] after the exposure with 100 µl supernatant of stimulated mononuclear cells. The supernatants (100 µl) collected from macrophage cultures 24 hrs after incubation was mixed with an equal volume of Griess reagent (Sigma-Aldrich, USA) and left for 10 min at RT. The absorbance of the reaction was measured at 540 nm in an ELISA reader as described elsewhere [35].

#### Estimation of cytokine levels- IFN-γ/IL-12/IL-10/IL-4 in lymphocytes of cured and endemic patients

PBMCs (1×10^6^ cells/ml) from human patients were plated in 96-well culture plates and the recombinant protein rLdHSP70 and its combination with rLdPDI, rLdTPI, and rLdEL-2 were added at a concentration of 10 µg/ml in triplicate wells. The level of IFN-γ, IL-12, IL-4 and IL-10 was estimated after 5 days of incubation with antigen using supernatants by enzyme-linked immunosorbent assay kit (OptEIA kit, BD Pharmingen, USA). The results were expressed as pg/ml. The lower detection limits for various cytokines were as follows: 5.1 pg/ml for IFN- γ, 30.8 pg/ml for IL-12, 5.1 pg/ml for IL-4 and 6.9 pg/ml for IL-10.

#### Quantification of mRNA cytokines and inducible NO synthase (iNOS) by quantitative real time PCR in vaccinated hamsters

qRT-PCR was performed to assess the mRNAs expression profile for various cytokines and iNOS in splenocytes of hamsters. Total RNA was isolated from splenic tissues using Tri-reagent (Invitrogen, USA) as described elsewhere [27]. One microgram of total RNA was used for the synthesis of cDNA using a first-strand cDNA synthesis kit (Fermentas, USA). For real-time PCR, primers were designed using Beacon Designer software (Bio-Rad, USA) based on the cytokines and iNOS mRNA sequences available on PubMed [36] (Table 1). qRT-PCR was performed as per the protocol described earlier by Samant et al [37]. Briefly, it was carried out with 12.5 µl of SYBR green PCR master mix, 1 µg of cDNA, and primers at a final concentration of 300 nM in a final volume of 25 µl. PCR was performed under the following conditions: initial denaturation at 95°C for 2 min followed by 40 cycles, each consisting of denaturation at 95°C for 30 s, annealing at 55°C for 40 s, and extension at 72°C for 40 s per cycle using the iQ5 multicolour real-time PCR system (Bio-Rad, USA). cDNAs from infected control hamsters were used as “comparator samples” for quantification of those corresponding to test sample. The normalization of all the quantifications was done by using housekeeping gene Hypoxanthine phosphoribosyl transferase (HPRT). A negative control (without c-DNA) was used to eliminate nonspecific reactions. The cycle threshold (CT) value was defined as the number of PCR cycles required for the fluorescence signal to exceed the detection threshold value (background noise). Differences in gene expression were calculated by the comparative CT method as described earlier [37] wherein test samples compared to a comparator sample. Results are expressed as the degrees of difference between ΔCT values of test and comparator samples.

**Table 1:** Sequences of forward and reverse primers of hamster cytokines used for quantitative real time PCR (qRT-PCR).

S.No.	Primer	Primer sequence
1	HGPRT FP	5′ GATAGATCCACTCCCATAACTG 3′
	HGPRT RP	5′ TACCTTCAACAATCAAGACATTC 3′
2	IFNγ FP	5′ GCTTAGATGTCGTGAATGG 3′
	IFNγ RP	5′ GCTGCTGTTGAAGAAGTTAG 3′
3	IL-12 FP	5′ TATGTTGTAGAGGTGGACTG 3′
	IL-12 RP	5′ TTGTGGCAGGTGTATTGG 3′
4	IL-4 FP	5′ GCCATCCTGCTCTGCCTTC 3′
	IL-4 RP	5′ TCCGTGGAGTTCTTCCTTGC 3′
5	IL-10 FP	5′ TGCCAAACCTTATCAGAAATG 3′
	IL-10 RP	5′ AGTTATCCTTCACCTGTTCC 3′
6	iNOS FP	5′ CGACGGCACCATCAGAGG 3′
	iNOS RP	5′ AGGATCAGAGGCAGCACATC 3′

#### Measurement of serum antibody response in vaccinated hamsters

The level of immunoglobulins, IgG and its isotypes in the serum sample of hamsters of different experimental groups was measured as per protocol by Samant et al [37] with slight modifications. Briefly, 96-well ELISA plates (Nunc, Denmark) were coated with 0.2 µg/100 µl/well recombinant proteins followed by incubation at 4°C and blocking with 1.5% BSA. Sera from different vaccinated groups were used at a dilution of 1/100 for IgG, IgG1, and IgG2 and kept for 2 h at RT. Biotin conjugated mouse anti-Armenian and Syrian hamster IgG, IgG1 and IgG2 (BD Pharmingen, USA) were added for 1 h at room temperature at 1/1000 dilutions and were further incubated with peroxidase-conjugated streptavidin at 1/1000 (BD Pharmingen, USA) for 1 h. Finally, the substrate O-phenylenediamine dihydrochloride (Sigma-Aldrich, USA) was added and the plate was read at 492 nm.

### Statistical analysis

Data were expressed as mean ± S.D. In each experiment 15–20 animals were used in each group. Two sets of experiments were performed independently and the results were analyzed by one-way ANOVA test using Prism Graphpad software program. The upper level of significance was chosen as P<0.001 (highly significant).

## Results

### LdHSP70 was cloned, sequenced and expressed in *E.coli Rosetta* strain

**Figure 1:**
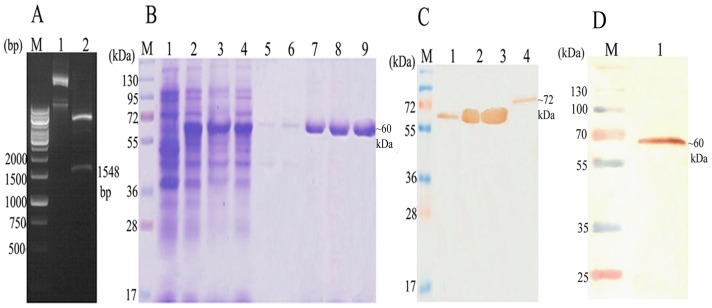
Molecular characterization of *L. donovani* Heat Shock Protein70 (LdHSP70). **(A)** Clone confirmation of LdHSP70 in pET28a^+^ vector. M: 1 kb molecular mass marker; Lane1: Undigested pET28a^+^-LdHSP70; Lane2: BamHI and EcoRI digested pET28a^+^-LdHSP70; **(B)** Expression, purification and elution of rLdHSP70 at 200 mM of imidazole concentration (conc.) and separation in 12%SDS PAGE. M: molecular weight markers, Lane 1: whole cell lysate (WCL) of uninduced *E.coli*; Lane 2: WCL of *E.coli* induced at 37°C and 1 mM IPTG; Lane 3: soup of protein; Lane 4: flow through fraction from column; Lane 5 and 6: wash fraction at 10 mM & 50 mM imidazole conc. respectively; Lane 7, 8 and 9: purified protein fractions; **(C)** Western blot analysis using anti rLdHSP70 antibody; M: molecular mass marker; Lane1: WCL without IPTG induction; Lane 2: WCL with IPTG (1 mM) induction at 37°C, Lane 3: purified protein; Lane 4: soluble *L. donovani*; **(D)** Western blot analysis of rLdHSP70 with patients serum. M: molecular mass marker; Lane 1: blot with rLdHSP70.

The LdHSP70 (1548 bp) gene of *L. donovani* was successfully cloned in T/A vector and sequenced. The homology of LdHSP70 has been found to the tune of 98% with *L. infantum* and *L. major* and 99% *with L. mexicana*. Consequently, LdHSP70 was further sub-cloned in bacterial expression vector pET28a^+^ (Fig. 1A), purified by affinity chromatography using Ni^2+^ NTA agarose beads containing columns and eluted at 250 mM imidazole concentration. The size of the eluted rLdHSP70 proteins was ∼60 kDa (Fig. 1B) which was further confirmed by western blot with anti-serum antibody raised in Swiss mice. Immunoblot analysis of SLD of *L. donovani* promastigote with the polyclonal anti-rLdHSP70 antibody detected the band beloning to 72 kDa in SLD (Fig. 1C). Western blot analysis of rLdHSP70 with patients sera also showed a dominant protein band of ∼60 kDa (Fig. 1D). The results showed that the recombinant was correctly constructed and expressed in bacterial expression vector.

### Combination of rLdHSP70 + rLdPDI stimulated optimum lymphoproliferative response and NO production in Cured *Leishmania* infected hamsters

**Figure 2:**
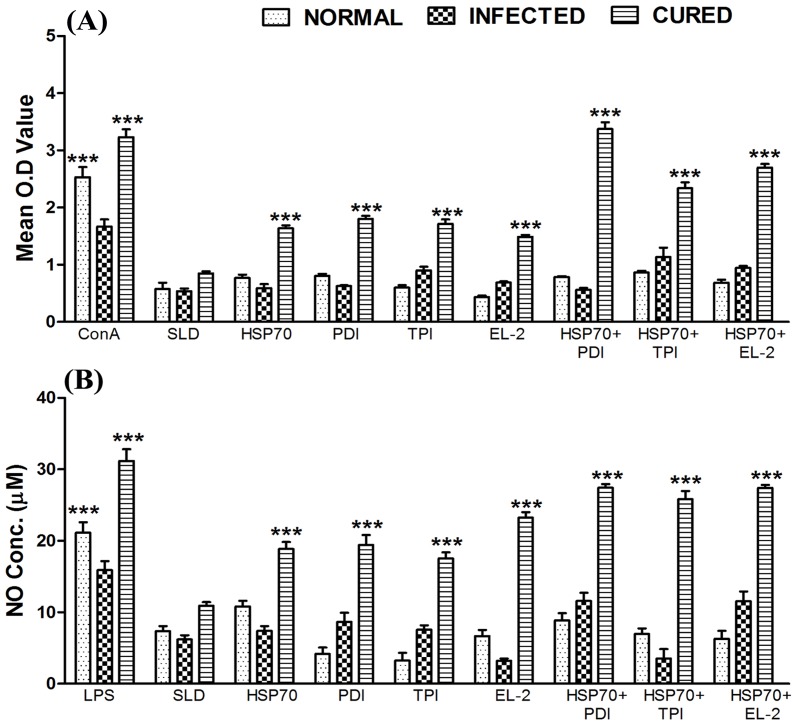
Cellular responses of the combinations rLdHSP70 + rLdPDI/rLdHSP70 + rLdTPI/rLdHSP70 + rLdEL-2 in hamsters lymphocytes (lymph node cell). **(A)**\ LTT response of mononuclear cells from normal, *L. donovani* infected (25 day p.c.) and treated hamsters in response to Con A, SLD and combinations (rLdHSP70 + rLdPDI/rLdHSP70 + rLdTPI/rLdHSP70 + rLdEL-2) at 10 µg/ml each. Proliferative response was represented as mean O.D of stimulated cells - mean O.D of unstimulated control cells. Each bar represents the pooled data (mean ± S.D. value) of 6 hamsters and the data represent the means of triplicate wells ± S.D. of each hamster as mean O.D of stimulated cells and mean O.D of unstimulated control cells. **(B)** **Nitric oxide production (µM)**: Peritoneal macrophages were stimulated with the supernatants of stimulated lymphocytes (3 days for mitogen and 5 days for test antigens) of normal, infected and cured hamsters in response to combinations with rLdHSP70, SLD and LPS respectively at 10 µg/ml each. The estimation of NO production was done using Greiss reagent in supernatants collected from macrophage cultures 24 h after incubation and the absorbance of the reaction product was measured at 540 nm. Significance values indicate the difference between the SLD and combination with rLdHSP70 stimulation (*, *p*<0.05, **, *p*<0.01; ***, *p*<0.001).

The cellular responses of lymph node cells of cured hamsters were assessed for lymphoproliferative response and NO production against the mitogens - ConA or LPS; and antigens – SLD as well as recombinant proteins (rLdHSP70, rLdPDI, rLdTPI, rLdEL-2) either alone or in combinations with rLdHSP70. The comparison of lymphoproliferative (LTT) response of cured lymphocytes was done with that of normal as well as *L. donovani* infected lymphocytes, which served as controls. The rLdHSP70 alone stimulated ∼2 fold better proliferative response than SLD in mononuclear cells of cured hamsters, while among the various combinations tested along with - rLdPDI, rLdTPI and rLdEL-2, stimulation with rLdHSP70 + rLdPDI induced optimum proliferative responses (∼4 folds) followed by rLdHSP70 + rLdEL-2 (∼3 folds) and rLdHSP70 + rLdTPI (∼2.8 folds) when compared with SLD (Fig. 2A). Moreover, when compared to rLdHSP70 alone the combination of rLdHSP70 + rLdPDI induced significant proliferative responses (∼2.2 folds) (p<0.001). On contrary, other recombinant proteins individually have also shown lesser LTT response when compared to various combinations tested (Fig. 2A). Similarly, rLdHSP70 + rLdPDI stimulated optimum NO production (∼1.5 folds) in peritoneal macrophages of cured hamsters (p<0.001) as compared to rLdHSP70 followed by rLdHSP70 + rLdTPI, and rLdHSP70 + rLdEL-2 (Fig. 2B).

### Combination of rLdHSP70 + rLdPDI as well as rLdHSP70 + rLdEL-2 stimulated PBMCs from *Leishmania* patients optimally to proliferate and to stimulate dominant Thl- cytokine release

**Figure 3:**
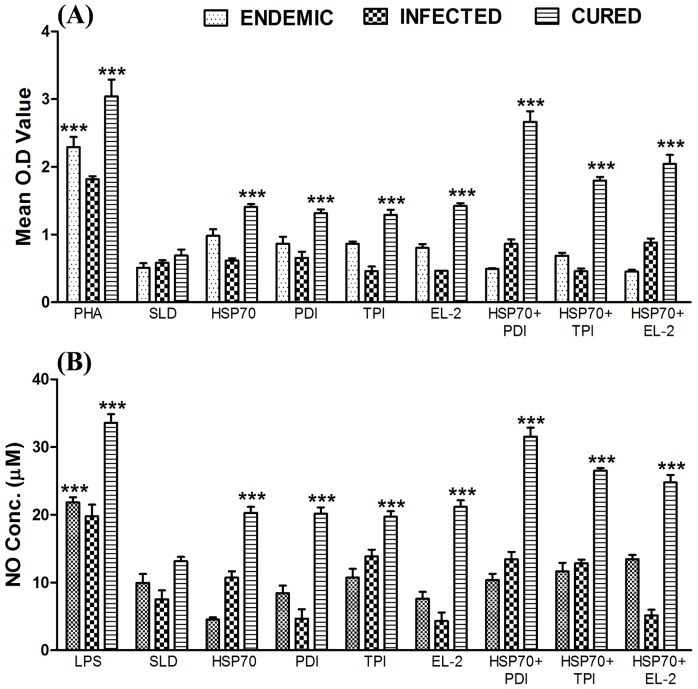
Cellular responses of the combinations rLdHSP70 + rLdPDI/rLdHSP70 + rLdTPI/rLdHSP70 + rLdEL-2 in patients sample. **(A)** LTT response of PBMC from *L. donovani* infected, endemic and cured patients in response to PHA, SLD and recombinant test antigens individually and in combination with rLdHSP70 10 µg/ml each. Proliferation was represented as mean O.D of stimulated cell - mean O.D of unstimulated control. Each bar represents the pooled data (mean ± S.D. value) of stimulated PBMCs of each group. **(B)** Significance values indicate the difference between the SLD and rLdHSP-70/rLdPDI/rLdTPI/rLdEL-2 and rLdHSP-70 + rLdPDI/rLdHSP-70 + rLdTPI/rLdHSP-70 + rLdEL-2 stimulation (*, *p*<0.05**, *p*<0.01; ***, *p*<0.001).

We further corroborated the proliferative responses and cytokine production of the various combinations as well as individual recombinant proteins in PBMCs of cured *Leishmania* infected patients. The comparison was made with the PBMCs from cured patients with *L. donovani* infected/endemic/normal groups. All the individuals in each study group were found to shown different responses. Endemic control and cured kala-azar patients groups have shown higher mean O.D value against PHA (2.291±0.159 and 2.807±0.275 respectively) as compared to unstimulated control. The combinations of rLdHSP70 + rLdPDI as well as rLdHSP70 + rLdEL-2 exhibited considerably better LTT response (∼3.2 and ∼3.0 folds respectively) as compared to SLD. Moreover, the two combinations stimulated considerably better LTT responses (∼1.5–2 folds) as compared to rLdHSP70, which was found statistically significant (p<0.001) in all the cured patients groups (Fig. 3A). In addition, the same combinations stimulated PBMCs of cured patients for higher NO production (∼2.4, and ∼2 folds respectively) as compared to SLD which was also significantly better as compared to rLdHSP70 (Fig. 3B).

**Figure 4:**
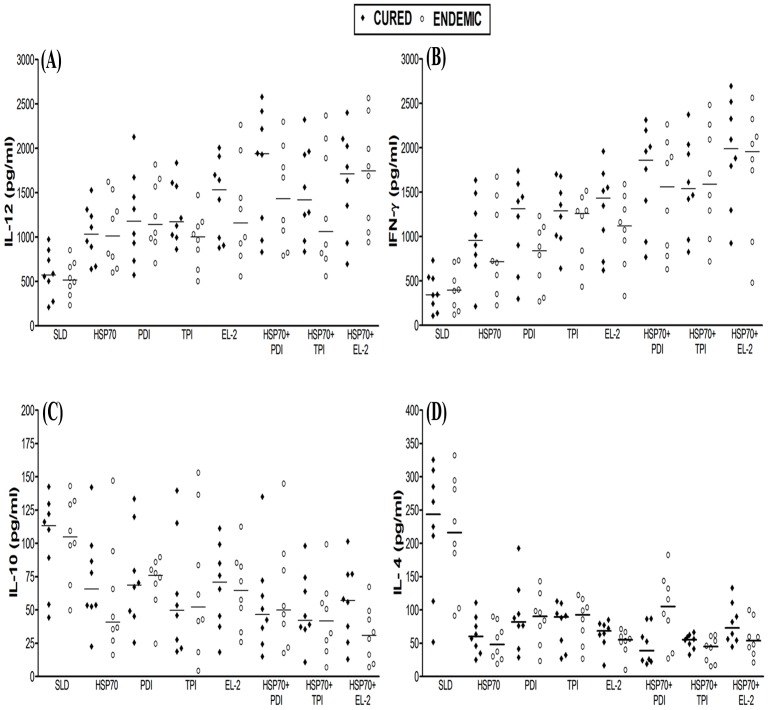
Cytokine production. **(A)** IL-12, **(B)** IFN-γ, **(C)** IL-10, and **(D)** IL-4, in PBMCs from individuals of cured VL patients (n = 8) and endemic controls (n = 8) in response to recombinant proteins individually as well as their combinations with rLdHSP70 and SLD antigens, each data point represents one individual. The results were expressed as picograms (pg) of cytokine/ml, based on the standard curves of the respective cytokine provided in the kit. The lower detection limits for various cytokines were as follows: for 5.1 pg/ml for IFN- γ, 30.8 pg/ml for IL-12, 5.1 pg/ml for IL-4 and 6.9 pg/ml for IL-10. Values are given as concentration in pg/ml.

We further assessed the production of Th1/Th2 cytokine levels in PBMCs from cured patients in response to the various combinations. The level of IL-12 and IFN-γ, the signatures of Th1 type of response, were noticed to be higher in the supernatant of cured patients against rLdHSP70 and the combinations with rLdHSP70. Though rLdHSP70 itself induced moderate level of IFN-γ response (∼2.8 folds) while in combinations, rLdHSP70 + rLdPDI and rLdHSP70 + rLdEL-2 induced maximum level of IFN-γ response (∼5.4 and ∼5.8 folds respectively) followed by rLdHSP70 + rLdTPI (∼4.4 folds) in comparison to SLD. The induction level of IFN-γ in response to the combinations, rLdHSP70 + rLdPDI and rLdHSP70 + rLdEL-2 was found to be ∼2 folds higher than rLdHSP70 itself. Similarly, it was observed that level of IL-12 was comparatively higher against rLdHSP70 + rLdPDI (∼4 folds) in comparison to SLD which was also higher (∼2 folds) as compared to rLdHSP70 alone (Fig. 4A&B). On the other hand, very low level of IL-4 cytokine was detected in supernatant of cured patients against rLdHSP70 + rLdPDI (∼6.2 folds) followed by rLdHSP70 + rLdTPI (∼4.4 folds) and rLdHSP70 + rLdEL-2 (∼3.4 folds) in comparison to SLD. However, the combination of rLdHSP70 + rLdPDI has shown higher level of IL-4 down regulation than rLdHSP70. Similarly, IL-10 level was also down regulated but its level was lower in comparison to IL-4. PBMCs of cured/endemic contacts generated a mixed type of Th1/Th2 cytokine response against SLD wherein high levels of IL-10 and IL-4 and comparatively low level of IFN-γ and IL-12 were noticed in response to rLdHSP70 (Fig. 4C & D). The result represents that recombinant Th1 protein in combination with rLdHSP70 performed as dominant T-cell antigens to which majority of the cured patients PBMCs have responded positively.

### Vaccination with rLdHSP70 + rLdPDI and rLdHSP70 + rLdEL-2 offered maximum protection against *L. donovani* challenge in Hamsters

**Figure 5:**
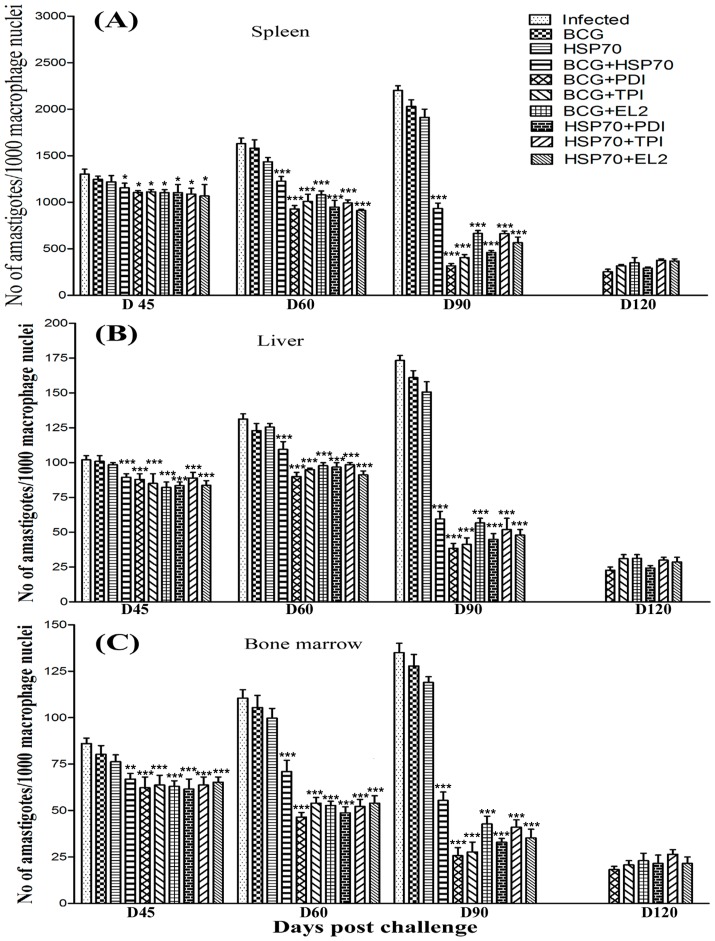
Prophylactic efficacy of combinations in hamsters against *L. donovani* challenge. *Leishmania* parasite burden (number. of amastigotes per 1000 cell nuclei) in the dab smears of spleen **(A)**, liver **(B)** and bone marrow **(C)** of hamsters on days 45, 60, 90, and 120 post challenge (p.c.) Since the non-vaccinated challenged (infected control) and the *Bacillus Calmette Guerin* (BCG) alone/rLdHSP70 alone vaccinated and challenged group died after day 90 (D90) of the study period, their corresponding bars are not depicted in A, B and C. Significance values indicate the difference between the vaccinated and infected group (*, p<0.05; **, p<0.01; and ***, p<0.001).

**Figure 6:**
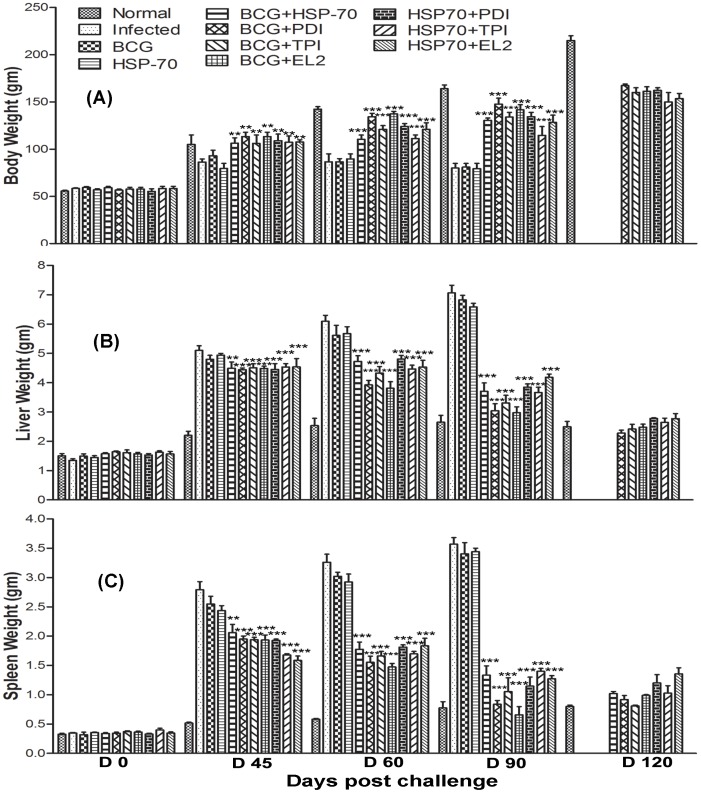
Prophylactic efficacy of combinations in hamsters against *L. donovani* challenge. Body weight **(A)**, liver weight **(B)** and spleen weight **(C)**, of hamsters in gram (gm) on days 0, 45, 60, 90 and 120 p.c. Since infected control, the BCG alone/rLdHSP70 alone vaccinated and challenged group died after day 90 (D 90) of the study period, their corresponding bars are not shown in A, B and C. Significance values indicate the difference between the vaccinated and infected group (*, p<0.05; **, p<0.01; and ***, p<0.001).

**Figure 7:**
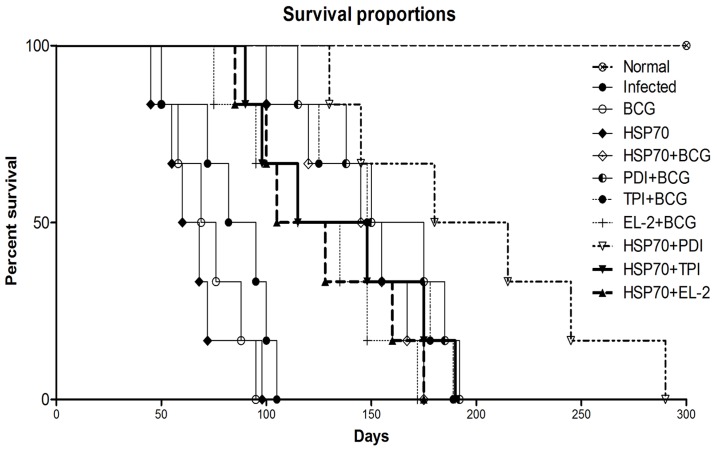
Survival curve analysis of different experimental groups. Survival of animals (6 hamsters in each experimental group) was observed up to day 300 p.c.

Since, *in vitro* studies on cured hamster's/patient's lymphocytes have revealed that Th1 stimulatory proteins - rLdPDI, rLdTPI and rLdEL-2, in combination with rLdHSP70 were able to generate a strong Th1-type immune response, we further evaluated the same combinations for their prophylactic efficacies in hamsters against *L. donovani* challenges. The animals vaccinated with the combinations viz. rLdHSP70 + rLdPDI, rLdHSP70 + rLdTPI and rLdHSP70 + rLdEL-2 have shown considerably good prophylactic efficacy to tune of 80%, 70%, and 74% respectively, against *L. donovani* challenges. On the other hand, when the recombinant proteins viz. rLdPDI, rLdTPI and rLdEL-2 were used along with BCG as an adjuvant for vaccination, the prophylactic efficacy was found to be similar as in combination with rLdHSP70. In general, all the vaccinated groups have shown decreased parasitic burden in spleen, liver and bone marrow onwards from day 45 to day 90 p.c. and was observed very low on day 90 p.c (p<0.001) (Fig 5A–C). Moreover, the hamsters vaccinated with all these combinations gained in body weight as compared to the control groups, when kept simultaneously up to day 90 post challenge (p.c.) (Fig. 6A). Further, an increase in the weight of spleen and liver was also observed in the above stated vaccinated animals. When hamsters were vaccinated with either rLdHSP70 or BCG, no reduction in parasite load was observed. However, the combination of rLdHSP70 + BCG has yielded moderate level (∼65%) of prophylactic efficacy. Moreover, all the vaccinated hamsters survived longer after the lethal challenge of *L. donovani* and remained healthy until the termination of the experiment 6–10 months post-infection. Among all the vaccinated groups, the hamsters vaccinated with rLdHSP70 + rLdPDI, survived and remained healthy till 10 months post-challenge (Fig. 7). Whereas, the hamsters of other vaccinated groups (except BCG or rLdHSP70 vaccinated and infected controls) could survive and remained healthy up to 6 months post-challenge (Fig. 7). On the other hand, there was progressive increase in parasite load in the hamsters immunized with either BCG or HSP70 alone as well as unimmunized ones and they succumbed to virulent *L. donovani* challenge within 2–3 months.

### Vaccination with the combinations, rLdHSP70 + rLdPDI, rLdHSP70 + rLdTPI, rLdHSP70 + rLdEL-2 stimulates *Leishmania*-specific cellular responses

**Figure 8:**
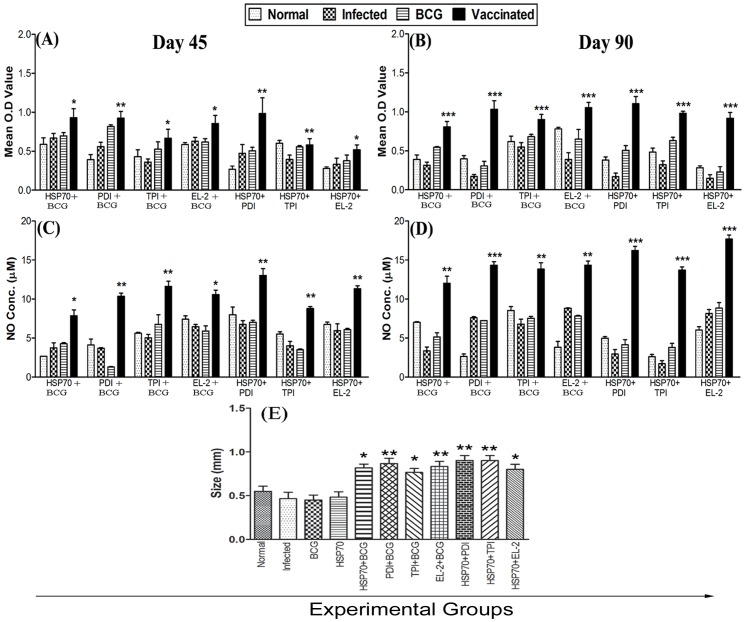
Cellular responses in vaccinated hamsters. LTT response (mean O.D value) to combinations and ConA in normal, infected control, BCG control, rLdHSP70 control groups as well as in group 5–11 vaccinated hamsters **(A)** on days 45, and **(B)** on day 90 p.c. Significance values indicate the difference between the vaccinated groups and infected group. **NO production** (µM) to LPS and combinations of rLdHSP70 in the naive macrophages co-incubated with supernatants of mononuclear cells isolated from respective group of vaccinated hamsters in comparison to the unimmunized infected controls, BCG immunized controls, rLdHSP70 control and uninfected normal hamsters **(C)** on day 45, and **(D)** on day 90 p.c. **DTH response** (mm) to soluble *L. donovani* (SLD) as footpad swelling on day 90 p.c **(E)**. Significance values indicate the difference between the vaccinated groups and infected group (*, p<0.05; **, p<0.01; and ***, p<0.001).

The cellular responses generated following vaccination with various combinations in hamsters and challenged with *L. donovani* were characterized. The LTT response of the lymph node cells from vaccinated hamsters upon stimulation with ConA was observed to be similar to those of the normal animals throughout the entire post challenged period (days 45, 60, 90 p.c.). In antigen-specific re-stimulation assays, the lymph node cells from hamsters vaccinated with the combinations viz. rLdHSP70 + rLdPDI, rLdHSP70 + rLdTPI and rLdHSP70 + rLdEL-2 showed an enhanced (∼6.5, ∼3.0 and ∼6.0 folds respectively) proliferative response than non-vaccinated infected control hamsters, on day 90 p.c.(p<0.001) (Fig. 8B). On the other hand, there was lesser proliferative response in animals vaccinated with BCG or rLdHSP70 alone as well as non-vaccinated infected control.

Since, generation of nitric oxide (NO) after macrophage activation by IFN-γ is an important factor in controlling leishmaniasis [38], the level of NO content in lymph node cells was measured in all the vaccinated hamsters and challenged with *L. donovani*. Hamsters vaccinated with all the three combinations viz. rLdHSP70 + rLdPDI, rLdHSP70 + rLdTPI, rLdHSP70 + rLdEL-2 showed an increase (∼6.0, ∼7.5 and ∼3.0 folds, respectively) in the level of NO production (p<0.001) on day 90 p.c (Fig. 8D) as compared to non-vaccinated infected control group of hamsters.

DTH is an index of cell mediated immunity *in vivo*. The hamsters vaccinated with various combinations elicited significant level of DTH response as compared to the other control groups on day 90 p.c. as compared to the infected control groups. However, all the hamsters vaccinated with various combinations have shown almost comparable DTH response (Fig. 8E).

### Vaccination with the combinations, rLdHSP70 + rLdPDI, rLdHSP70 + rLdTPI, rLdHSP70 + rLdEL-2 favours Th1-type cytokine responses as determined by real-time PCR

**Figure 9:**
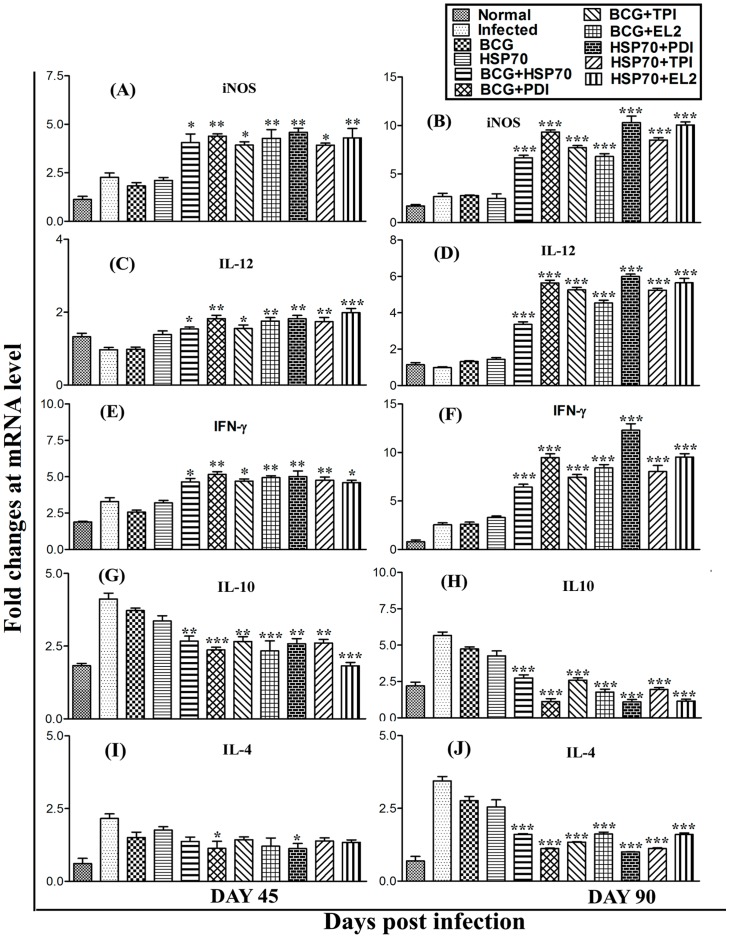
Splenic iNOS and cytokine (Th1/Th2) mRNA expression profile analysis of normal, infected, BCG or HSP70 control and vaccinated hamsters (n = 3/4) on day 45 and 90 p.c. by qRT-PCR. Splenic inducible nitric oxide synthase (iNOS) **(A and B)** and cytokines, IL-12 **(C and D)**, IFN-γ **(E and F)**, IL-10 **(G and H)** and IL-4 **(I and J)** mRNA expression profile was assessed by qRT-PCR in all the experimental groups of hamsters on days 45 and 90 p.c. Data are presented as means ± S.D and are representative of two independent experiments with similar results. Significance values indicate the difference between the vaccinated and infected group (*, p<0.05; **, p<0.01; and ***, p<0.001).

Since, cured *Leishmania* infected patients PBMCs elicited Th1-type of immune responses against all the combinations viz. rLdHSP70 + rLdPDI, rLdHSP70 + rLdTPI, rLdHSP70 + rLdEL-2 we further assessed the cytokine responses in hamsters vaccinated with these combinations. The mRNA expression level of the Th1 and Th2 cytokines in vaccinated group of hamsters was estimated by qRT-PCR, on days 45 and 90 p.c. A moderate increase (∼2 to ∼3 folds) in expression levels of iNOS mRNA transcripts, which is known to impart major role in control of leishmaniasis, was observed in all the hamsters vaccinated with above mentioned combinations on day 45 p.c., which was further increased significantly (∼3 to ∼4 folds) on day 90 p.c., as compared to non-vaccinated infected control (Fig. 9A&B). Similarly, one of the healing inflammatory cytokine IL-12 was found to be significantly up-regulated upto ∼8 folds on day 90 p.c. (p<0.001) in rLdHSP70 + rLdPDI vaccinated hamsters followed by rLdHSP70 + rLdEL-2 vaccinated (∼6.5 folds), as compared with infected control (Fig. 9C&D). At the same time point, the expression of IFN-γ was also higher (∼5 folds) in rLdHSP70 + rLdPDI vaccinated hamsters followed by vaccination with rLdHSP70 + rLdEL-2 (∼3.5 folds) in comparison to *L. donovani*-infected group (Fig. 9E&F). On the contrary, these Th1 cytokines were down-regulated in infected control groups whereas, Th2 cytokines viz. IL-4 and IL-10 were significantly up-regulated in infected control groups, indicating progressive VL whilst these cytokines were highly down-regulated in vaccinated animals reflecting the situation in cured VL (Fig. 9G–J).

### Serological responses (*Leishmania*-specific IgG and its isotypes) in vaccinated hamsters

**Figure 10:**
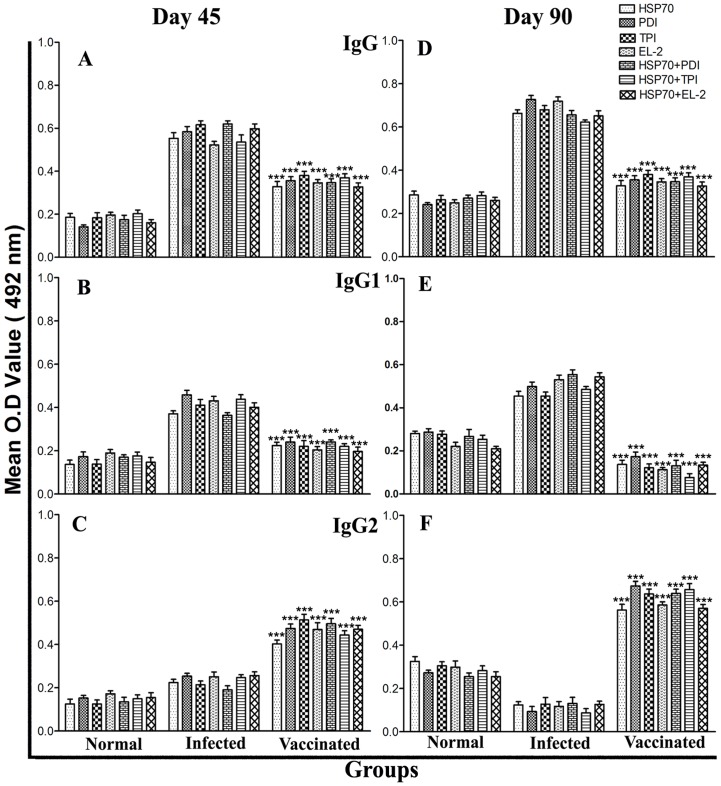
Antibody response in vaccinated hamsters. IgG (A and D) and its isotypes IgG1 (B and E) and IgG2 (C and F) in vaccinated hamsters in comparison to the non-vaccinated infected hamsters on days 45 and 90 p.c. Serum samples were collected from different experimental groups of hamsters at designated time points and assayed for specific IgG, IgG1, and IgG2 levels by ELISA. Data are presented as the absorbance at 492 nm and are means ± SD for 3–4 hamsters per group in triplicate wells. Data are representative of two independent experiments with similar results. Significance values indicate the difference between the vaccinated and infected group (*, p<0.05; **, p<0.01; and ***, p<0.001).

We further checked for antibody levels in the serum of vaccinated animals in comparison to infected control animals. Although it is well known that the cytokines such as IFN-γ and IL-4 regulate immunoglobulin class switching of IgG2 and IgG1 respectively, in this study the level of IgG2 was found elevated up to ∼5 folds in animals vaccinated with rLdHSP70 + rLdPDI, rLdHSP70 + rLdTPI, rLdHSP70 + rLdEL-2, BCG + rLdPDI, BCG + rLdTPI and BCG + rLdEL-2 over the others on day 90 p.c., which is indicative for Th1-type immune response (Fig. 10F). Higher levels of IgG1 were observed in the infected control animals. As a measure of CMI, the elevated level of IgG2 was consistent with the development of effective immune responses.

## Discussion

During the course of infection of mammalian hosts with either intracellular or extracellular pathogens, HSPs become most important targets of the immune response [39]–[44]. In case of intracellular organisms, particularly, *Leishmania* spp., *Plasmodium* spp., *Trypanosoma cruzi*, and *Mycobacterium spp*., immune responses to HSP at both B- and T- cell levels have been described [39], [41], [42], [44]. Further, HSP mediate essential cytoprotective function, like production of cytokines, provide maturation signals and facilitate peptides to antigen presenting cells through receptor-mediated interactions. Therefore, all these functions favor HSPs as potential immunoregulatory agents and thus allow their exploitation for the development of a new generation therapeutic and prophylactic vaccine against infectious diseases [45]–[48]. Among the various studied HSP families, the HSP70 family is well characterized and has attracted much attention because of its versatile functions in the immune system. It is considered as the ‘workhouse’ of the chaperons, because of its promiscuity to assist in folding new polypeptide chains [49]–[51]. Besides the chaperone activity, HSP70 molecules can function as an adjuvant also [52], [53]. The use of novel vaccine adjuvants or carrier proteins, which are known to enhance the immunogenicity of the subunit antigens and provide T-cell help, can improve the potency of vaccines. The potential of HSP70 to function as adjuvants when fused to or co-delivered with protein antigens, make them attractive vaccine candidates [22], [24], [54]–[57].

The cure of VL is always associated with the induction of Th1 cytokines dominated by IFN-γ. Based on this fact, our earlier studies showed that a sub-fraction of *L. donovani* soluble protein ranging from 89.9 to 97.1 kDa, to be T-cell stimulatory and protective against experimental VL [12]–[14]. This sub-fraction, when subjected to proteomic analysis, revealed several Th1 stimulatory proteins including HSP70. Bhowmick et al. [26] and Kaur et al [23], [24] have earlier evaluated native HSP70 protein of *L. donovani*, for its immunogenicity and protective potential using electro-elution method. Herein, we successfully cloned, expressed and purified the HSP70 protein of *L. donovani* in order to obtain protein in large quantity for immunological characterization. In another study, cloning, expression and purification of *L. donovani* specific 70 kDa protein was done for the purpose of serodiagnosis of VL wherein the primers were designed on the basis of sequence of the peptide, identified from the immunogenic protein fraction corresponding to a HSP of MW 70 kDa containing 653 amino acids through MALDI TOF analysis [58], [59]. In our study, the HSP70 gene sequence analysis of *L. donovani* through nBLAST showed ∼98% identity with *L. major* and *L. infantum* and ∼99% homology with *L. mexicana*. Similarly, a comparative sequence analysis with human HSP70 revealed ∼72% homology.

It is well-known that a Th1 response is generated with increased production of IL-12 and IFN-γ and increased level of NO synthase in cured patients who has become immune to re-infection with *Leishmania* [7], [8], [34], [60], [61]. Therefore, the antigens that are involved in the stimulation of Th1 cell and initiation of memory Th1 cells can be considered for suitable vaccine candidate against VL. Further, unlike rodents, particularly mice, human VL has been demonstrating both Th1 and Th2 type immune responses, where protective immunity is achieved by polarization of immune response towards Th1 [62]. The clinicopathological profile of hamsters infected with *L. donovani*, however, closely mimics to human VL resulting in a persistently increasing parasite burden, progressive cachexia, hepato-splenomegaly, pancytopenia, hypergammaglobulinemia and ultimately death [30], [36], [63], [64]. Since, HSP70 was identified as Th1 stimulatory protein, we therefore, evaluated rLdHSP70 of *L. donovani* for its ability to stimulate cell proliferation and cytokine production on PBMCs from cured hamster as well as cured kala-azar patients infected with *L. donovani* and compared it with SLD. In addition, since, many workers have explored the adjuvant property of HSP70 which elicit strong humoral and cellular immune response when it is either fused or mixed with other antigens [22]–[24], [57], [65]–[71], the activity of rLdHSP70 was also simultaneously evaluated in combination with the potential Th1 proteins (rLdPDI, rLdTPI and rLdEL-2) identified in our laboratory [12]. rLdHSP70 stimulated significant lymphoproliferative response in cured hamsters/human as compared to SLD. It further induced optimum proliferative response when combined other Th1 proteins- rLdPDI, rLdTPI and rLdEL-2 wherein, among the three the combination of rLdHSP70 + rLdPDI exhibited better cellular responses. Further, since, in human as well as in experimental leishmaniasis, immunity is primarily mediated by T lymphocyte [72] which contributes in the immune response to *L. donovani* infection by producing cytokines which seems to be essential mediators of immunity to *Leishmania* [9], we characterized the cytokine responses in cured *Leishmania* patients/endemic contacts following stimulation with rLdHSP70 alone or in combinations with rLdPDI, rLdTPI and rLdEL-2. All the stimulants showed considerably good Thl cytokine responses (IFN-γ and IL-12) and downregulated Th2 cytokine responses (IL-10 and IL-4) when compared to rLdHSP70 alone. This may be attributed due to production of high amounts of IFN-γ by cured patients which in turn inhibit the production of IL-10 [73]. The study corroborates the findings of Skeiky et al [74] wherein recombinant HSP70 was effective in stimulating PBMCs from *L. braziliensis* infected individuals to proliferate and produced IFN-γ.

Encouraged with the observations made by *in vitro* study, we further evaluated the protective efficacy of all the three combinations in hamsters against *L. donovani* challenge. We noticed that on day 90 p.c, though, all the three combinations - LdHSP70 + rLdPDI, rLdHSP70 + rLdTPI and rLdHSP70 + rLdEL-2 offered resistance to *L. donovani* challenge to the tune of 70 to 80% in hamsters, the most effective combination was LdHSP70 + rLdPDI (80%). Moreover, among the vaccinated groups, LdHSP70 + rLdPDI vaccinated animals survived longer to the lethal challenge and remained healthy upto 10 months whereas the rLdHSP70 + rLdTPI and rLdHSP70 + rLdEL-2 vaccinated animals could survive upto six months only. This finding was similar when compared with the animal groups receiving recombinant proteins along with BCG as adjuvant, indicating the adjuvant/immunomodulatory like property of rLdHSP70.

Since, the immunogenicity of most antigens is highly limited in its pure form, it is often necessary to add adjuvant in order to achieve the desired immune responses (cellular and humoral immune response) for the control of disease progression. Further, it is well known that adjuvant stimulates the appropriate T-cell immunity for intracellular pathogens and HSPs are among the molecules able to stimulate antigen-presenting cells to secrete pro-inflammatory cytokines and strongly promote Th1 responses [75]–[77]. Moreover, it can stimulate production of NO by macrophages and up-regulation of co-stimulatory molecules that are required for priming naive T-cells. Therefore, the independent immunostimulatory capacity of HSPs makes them an attractive agent for the induction of specific immune responses as was observed in our study. Increasing evidences have indicated important roles of HSP70s, serving as carriers for antigens and promoting the induction/release of cytokines by different immune cells [53], [78]–[80]. Earlier, several workers already explored the adjuvant property of HSP70 molecule, eliciting strong humoral and cellular immune response when used in combination with various antigens in *Leishmania* parasite [22]–[24], [57], [65], [68]–[71], [81]. In the present study, we have also exploited the adjuvant property of LdHSP70 alongwith the potential Th1-stimulatory proteins - rLdPDI, rLdTPI and rLdEL-2 proteins and checked whether it can further enhance their immunogenicity and protective responses.

Given that the development of strong cellular immune responses like T-cell responses, NO production and DTH response imparts major role in protection against parasite and is also supposed to contribute to healing in VL [64], [82]–[86]. All the hamsters vaccinated with various combinations of rLdHSP70 and challenged with *L. donovani* exhibited enhanced proliferative response than in non-vaccinated infected control animals. Further, lymphocytes from all the vaccinated hamsters produced remarkably better level of NO in the macrophages of naive hamsters which also supported the view regarding the up-regulation of iNOS by Th1 cell-associated cytokines and confirm that the NO-mediated macrophage effector mechanism is critical in the control of parasite replication in the animal model [87], [88]. Further, as earlier reports have shown that successful vaccination of humans and animals is often related to antigen-induced DTH responses *in vivo* [36], [89], in the present study, hamsters vaccinated with all the three combinations have shown significant DTH response as compared with other control groups which can be correlated with disease progression in hamsters. Moreover, since, it has been well established that the resistance against leishmaniasis is conferred by Th1 cells while susceptibility is conferred by Th2 cells, these cytokines were further checked by qRT-PCR. It was observed that the vaccination of hamsters with all the three combinations resulted in significant up-regulation of Th1 type of immune response by day 90 p.c. The mRNA transcript level of IFN-γ, a signature cytokine of the Th1-type response, was found to be highly up-regulated, particularly in LdHSP70 + rLdPDI vaccinated animals (∼5 folds), over the infected control groups. Similarly, the level of IL-12 was also elevated upto ∼8 folds in LdHSP70 + rLdPDI vaccinated animals and were highly down-regulated in infected animals. However, there was no increase in the level of key macrophage deactivating Th2 cytokines- IL-10 and IL-4, which have a definitive association with an acute phase of VL [36], [90]. Moreover, the levels of IgG and IgG1 antibodies, whose increase is directly proportional to the *L. donovani* loads [91], was present at very low level in the serum samples of vaccinated animals whereas the IgG2 titres were the highest in the vaccinated animals and these observations were consistent with the development of effective immune responses [37].

The major observation from this study was that *L. donovani* rLdHSP70, yielded a specific Th1-like immune response in lymphocytes of cured hamsters/cured patients and it induced even stronger cellular response when was combined with all the three Th1 stimulatory proteins. Moreover, all the three combinations generated more or less similar immunoprotective response, however, depending on the survival data, LdHSP70 + rLdPDI appeared to be slightly better than the rLdHSP70 + rLdTPI and rLdHSP70 + rLdEL-2. Microbial HSP have been implicated in the induction of the innate and adaptive arms of the immune response [92], [93]. Among the various HSPs, HSP70 is one of the most abundant chaperones therefore the additive effect may be due to its chaperone activity.

There has been various HSP70-based vaccine strategies described to enhance fusion vaccine potency [94], as it has strong ability to complexes with antigenic peptides and can generate a peptide-specific cellular immune response more efficient than the peptide alone [93]. Since, HSP70 has been identified as one of the Th1-stimulatory protein of *Leishmania* parasites, it could act as a strong potentiator of the immune system and can be a better alternative of BCG as an adjuvant which has several side effects. Thus, it may be surmised that vaccines based on combination of *Leishmania* HSP70 with Th1-stimulatory proteins can be of relevance for future vaccination strategies against this intracellular pathogen.
